# Deletion of the Basement Membrane Heparan Sulfate Proteoglycan Type XVIII Collagen Causes Hypertriglyceridemia in Mice and Humans

**DOI:** 10.1371/journal.pone.0013919

**Published:** 2010-11-10

**Authors:** Joseph R. Bishop, Maria Rita Passos-Bueno, Loren Fong, Kristin I. Stanford, Jon C. Gonzales, Erika Yeh, Stephen G. Young, Andre Bensadoun, Joseph L. Witztum, Jeffrey D. Esko, Karen S. Moulton

**Affiliations:** 1 Department of Cellular and Molecular Medicine, University of California San Diego, La Jolla, California, United States of America; 2 Human Genome Center, Department of Genetics and Evolutionary Biology, Institute of Biosciences, University of São Paulo, São Paulo, São Paulo, Brazil; 3 Division of Cardiology, David Geffen School of Medicine, University of California Los Angeles, Los Angeles, California, United States of America; 4 Division of Nutritional Sciences, Cornell University, Ithaca, New York, United States of America; 5 Department of Medicine, University of California San Diego, La Jolla, California, United States of America; 6 Division of Cardiology, University of Colorado Denver, Aurora, Colorado, United States of America; Maastricht University, Netherlands

## Abstract

**Background:**

Lipoprotein lipase (Lpl) acts on triglyceride-rich lipoproteins in the peripheral circulation, liberating free fatty acids for energy metabolism or storage. This essential enzyme is synthesized in parenchymal cells of adipose tissue, heart, and skeletal muscle and migrates to the luminal side of the vascular endothelium where it acts upon circulating lipoproteins. Prior studies suggested that Lpl is immobilized by way of heparan sulfate proteoglycans on the endothelium, but genetically altering endothelial cell heparan sulfate had no effect on Lpl localization or lipolysis. The objective of this study was to determine if extracellular matrix proteoglycans affect Lpl distribution and triglyceride metabolism.

**Methods and Findings:**

We examined mutant mice defective in collagen XVIII (Col18), a heparan sulfate proteoglycan present in vascular basement membranes. Loss of Col18 reduces plasma levels of Lpl enzyme and activity, which results in mild fasting hypertriglyceridemia and diet-induced hyperchylomicronemia. Humans with Knobloch Syndrome caused by a null mutation in the vascular form of Col18 also present lower than normal plasma Lpl mass and activity and exhibit fasting hypertriglyceridemia.

**Conclusions:**

This is the first report demonstrating that Lpl presentation on the lumenal side of the endothelium depends on a basement membrane proteoglycan and demonstrates a previously unrecognized phenotype in patients lacking Col18.

## Introduction

Heparan sulfate proteoglycans present in basement membranes aid in the formation of tissue barriers and facilitate cell adhesion and migration during development [1]. The major basement membrane heparan sulfate proteoglycans include collagen XVIII (Col18) and perlecan found ubiquitously in the vascular basement membrane zones of tissues, and agrin, a critical component of the neuromuscular junction basement membrane [2], [3], [4]. Systemic loss-of-function alleles in perlecan and agrin are lethal in mice [5], [6], [7]. In contrast, Col18-deficient mice (*Col18a1* ^−/−^) are viable but exhibit major ocular defects including abnormal retinal vessel development and poor anchoring of vitreal collagen fibrils to the inner lining membrane of the retina [8], [9], [10]. Col18-deficiency also leads to increased vascular permeability of large and small blood vessels and thickening of basement membranes in the heart, kidney, and skin [9], [11].

Lipoprotein lipase (Lpl) hydrolyzes triglycerides in triglyceride-rich lipoproteins in the circulation, liberating free fatty acids and monoacylglycerol for storage and energy production. This essential enzyme is synthesized in parenchymal cells of adipose tissue, heart, and skeletal muscle and migrates to the luminal side of the vascular endothelium where it acts upon circulating lipoproteins [12]. Recent studies indicate that Lpl is tethered to the lumenal side of vascular endothelium by Gpihbp1, a protein found on the surface of endothelial cells in the adipose, heart, and skeletal muscle [13], [14]. Genetic ablation of *Gpihbp1* reduces vascular presentation of Lpl and plasma levels of Lpl activity and mass, which causes striking hypertriglyceridemia. Injection of heparin releases Lpl into the circulation and rescues the hyperlipidemic phenotype in *Gpihbp1*-deficient mice; however, both Lpl release and the subsequent decline of plasma triglycerides occur more slowly in the mutant compared to wild-type animals [15]. These results suggest that Lpl is stored in a subendothelial compartment awaiting transport. Recent findings demonstrate that Gpihbp1 also facilitates the transport of Lpl from subendothelial compartments to the lumenal side of the vasculature [14].

Lpl binds to heparin, suggesting that it might associate with heparan sulfate that is covalently attached to extracellular matrix or cell surface proteoglycans [16], [17]. Mutations in lipoprotein lipase that impair heparin-binding reduce enzyme stability and causes abnormalities in lipid delivery to tissues [18]. However, genetically altering heparan sulfate selectively in endothelial cells does not affect plasma levels of Lpl or lipolysis [15], consistent with the hypothesis that the heparin-binding site of Lpl also interacts with acidic domain in Gpihbp1 [19]. In this work, we found that deletion of the heparan sulfate proteoglycan Col18 resulted in reduced vascular Lpl mass and activity in mice and caused mild hypertriglyceridemia, suggesting that the relevant Lpl-heparan sulfate proteoglycan interaction occurs in the subendothelial space. In contrast, mutant mice lacking the heparan sulfate attachment sites in perlecan (*Hspg2^Δ3/Δ3^*) did not have mild hypertriglyceridemia. These findings are of clinical relevance because we show that patients lacking the primary vascular form of Col18 (Knobloch Syndrome, OMIM 267750) also exhibit fasting hypertriglyceridemia and decreased plasma Lpl, previously unrecognized phenotypes in these patients.

## Results

### Col18-deficiency causes hypertriglyceridemia in mice

**Figure 1:**
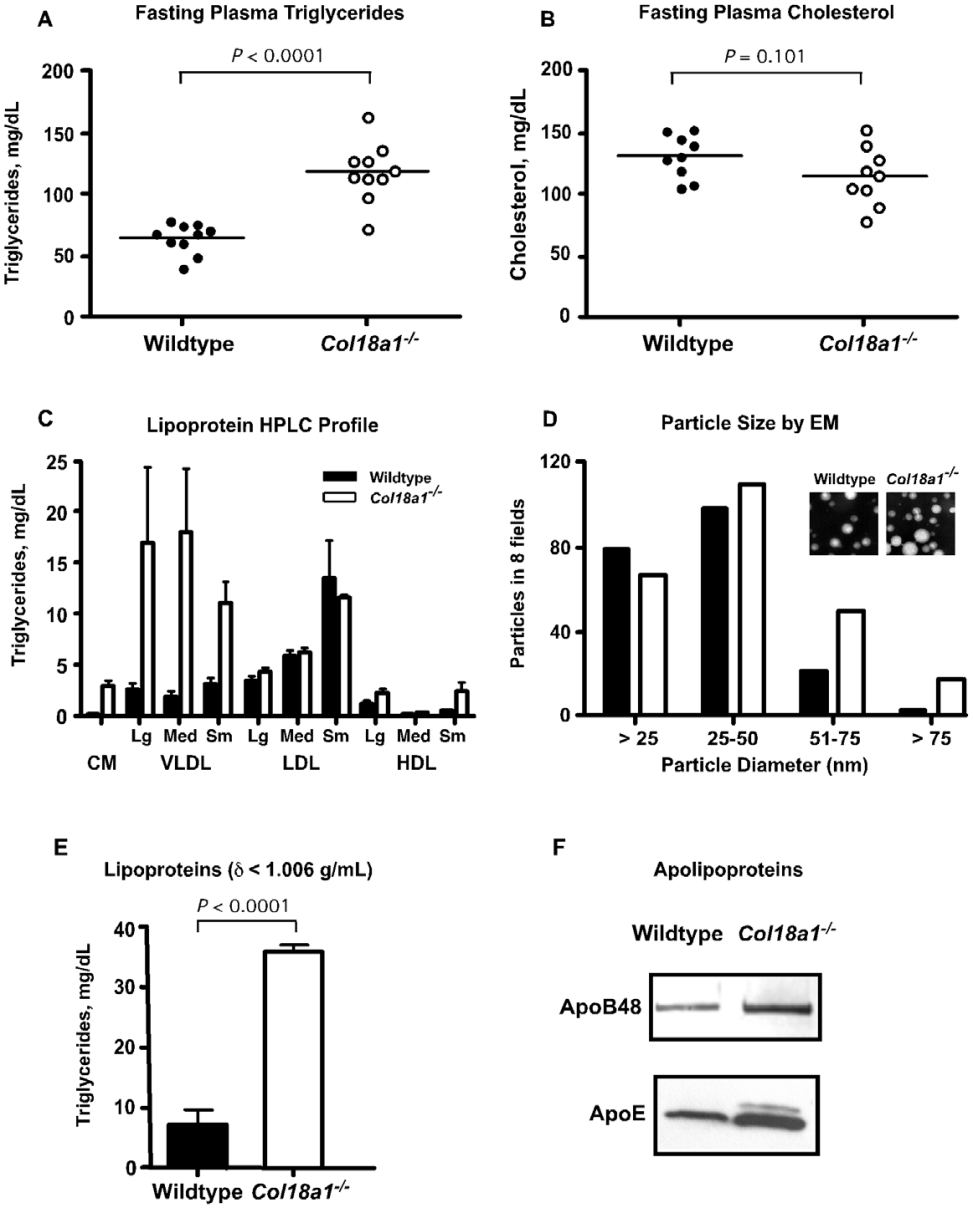
Hypertriglyceridemia in fasted *Col18a1^**−/−**^* mice. (**A**) Retro-orbital sinus blood was taken from wild-type and *Col18a1^−/−^* mice after fasting the animals for 4 hrs in the morning. Total plasma triglycerides were compared between mutant and wild-type (n = 10 mice, unpaired t-test, *P*<0.0001) and results were repeated. (**B**) Plasma cholesterol was analyzed in the same samples. (**C**) Triglyceride distribution across plasma lipoprotein subclasses. Plasma was pooled from 4 mice of each genotype and triglycerides in lipoprotein size classes were determined by LipoSEARCH FPLC. Control mice, filled bars; *Col18a1^−/−^* mice, open bars. Chylomicron (CM) >80 nm particle diameter; VLDL, 30–80 nm; LDL, 16–30 nm; and HDL, 8–16 nm. The experiment was performed twice and the error bars represent the range in the recovery of triglycerides in the two experiments. (**D**) Equal volumes of lipoproteins of δ<1.006 g/ml (pooled from n = 3 mice of each genotype) were analyzed by negative staining transmission electron microscopy (inset, 20,000× magnification, bar = 50 nm). The diameters of particles present in eight fields were analyzed in a blinded fashion. The experiment was repeated twice with comparable results. (**E**). The fraction of fasting lipoproteins of δ<1.006 g/ml from *Col18a1* mutants contained >4-fold more triglyceride than in wild type. The experiment was performed on pooled plasma samples from n = 4 mice and repeated three times. (**F**) Equal volumes of δ<1.006 g/ml lipoproteins were concentrated by membrane filtration and apolipoproteins were resolved by gradient SDS-PAGE, then visualized by Western blotting with rabbit anti-mouse apolipoprotein B-48/B-100 and apolipoprotein E (apoE) polyclonal antibodies.

To investigate the possibility that sub-vascular heparan sulfate proteoglycans participate in lipoprotein metabolism, we examined *Col18a1^−/−^* mice and *Hspg2^Δ3/Δ3^* mutant mice that lack the heparan sulfate attachment sites in the basement membrane proteoglycan, perlecan [20]. *Col18a1^−/−^* mice [8] had elevated plasma triglycerides compared to wild-type littermate controls (Fig. 1A) (65±13 mg/dl in control mice *vs*. 119±25 mg/dl in mutants, n = 10, *P*<0.0001). These values did not vary significantly according to the duration of fasting (4–8 hr), age of the animals (2–12 months) or their sex. Plasma cholesterol levels were slightly lower in mutants, but the difference did not achieve statistical significance (Fig. 1B) (132±17 mg/dl in wild type *vs*. 115±24 in mutants, n = 10, *P* = 0.101). *Col18a1* ^+/−^ heterozygotes did not display any changes in plasma lipids compared to the wild-type controls (data not shown). In contrast to *Col18a1* ^−/−^ mice, *Hspg2^Δ3/Δ3^* mice did not exhibit any change in fasting plasma triglycerides and cholesterol compared to wild type (plasma triglycerides, 44±15 mg/dL, n = 11 vs. 48±4 mg/dL, n = 12; plasma cholesterol levels, 69±8 mg/dL vs. 71±3 mg/dL), consistent with the studies in *Hspg2^Δ3/Δ3^ apoE^−/−^* mutants by Tran-Lundmark et al [21].

Analysis of the plasma lipoproteins in fasted *Col18a1* ^−/−^ animals by LipoSEARCH profiling, which uses curve fitting algorithms to gel filtration profiles to determine the relative amounts of lipoprotein subclasses, showed that the mutant accumulated large diameter particles corresponding in size to chylomicron remnants and VLDL lipoprotein subclasses (Fig. 1C). Electron microscopy showed significant enrichment of large diameter lipoproteins in the mutant (>50 nm, Fig. 1D). The plasma triglycerides floated at a δ<1.006 g/ml (Fig. 1E), and contained apolipoprotein B-100 (apoB-100), apolipoprotein B-48 (apoB-48) and apolipoprotein E (apoE) by western blotting (Fig. 1F), consistent with their identification as VLDL or chylomicron remnants.

### Delayed lipolysis in *Col18a1* ^−/−^ mice

**Figure 2:**
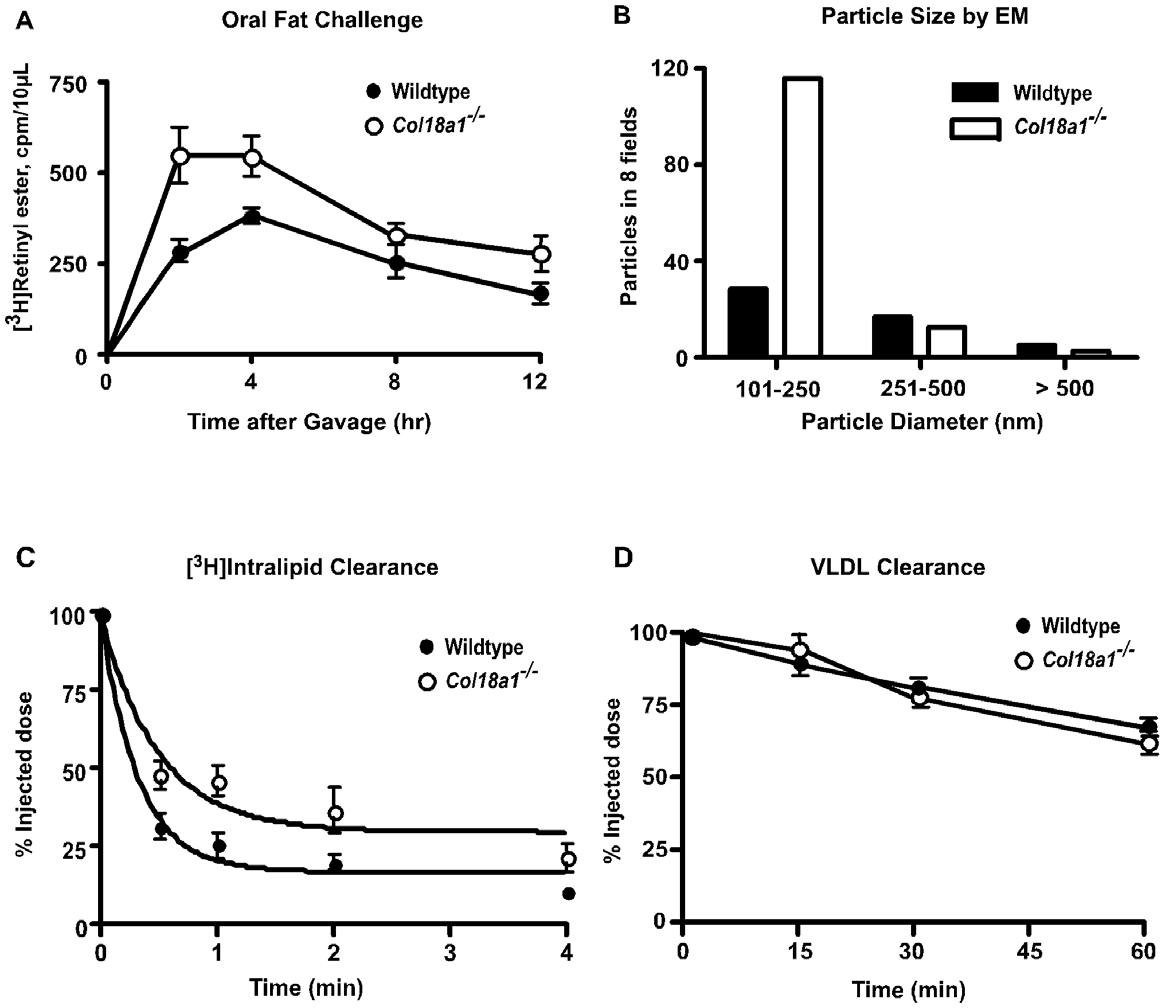
Characterization of hyperchylomicronemia in *Col18a1^**−/−**^* mice. (**A**) Chylomicron clearance assessed by retinyl ester excursion. Fasted wild-type (closed circles, *n* = 4) and *Col18a1^−/−^* mice (open circles, *n* = 4) were given 200 µl of corn oil containing [^3^H]retinol by oral gavage. Blood samples were taken at the indicated times and radioactivity in 10 µL of serum was determined by scintillation counting in triplicate. The values are expressed as mean ± SD and are representative of three separate experiments. (**B**) Equal volumes of δ<1.006 g/ml lipoproteins were purified from plasma collected from n = 3 mice of each genotype at 2 hrs post gavage, which corresponded to the time when the difference in [^3^H]-retinyl esters were maximal between *Col18a1^−/−^* and wild-type mice. The δ<1.006 g/ml lipoproteins were negative-stained and imaged by electron microscopy (20,000× magnification) and, the diameters of all particles in 8 fields were measured in a blinded fashion. The percentage of lipoproteins >100 nm in diameter were 2.8-fold increased in *Col18a1^−/−^* compared to wild-type mice. (**C**) [^3^H]Intralipid particles were injected intravenously in *Col18a1^−/−^* mice (open circles, n = 3) and wild-type mice (closed circles, n = 3) and blood was drawn from the retro-orbital sinus at the indicated time. Results are expressed as the percentage of cpm recovered after injection and are representative of two separate experiments. (**D**) Turnover of VLDL was measured in *Col18a1^−/−^* mice (open circles, n = 4) compared to controls (closed circles, n = 4). Human VLDL was injected intravenously and blood was drawn from the retro-orbital sinus at the indicated times. Human apoB-100 was measured by ELISA using MB47, which binds human but not murine apoB-100, exactly as described in [24]. Results are expressed as a percentage of the injected dose. The values are representative of two separate experiments.

To determine if turnover of dietary lipids was affected, we examined the rate of lipoprotein clearance after oral gavage with corn oil containing [^3^H]retinol, which is converted into fatty acylated retinyl esters and packaged into chylomicrons. After 2 hours, plasma levels of [^3^H]retinyl esters were elevated >2-fold in *Col18a1^−/−^* mice compared to wild-type animals and remained elevated for 4 hours (Fig. 2A). Thereafter, the rate of disappearance of ^3^H-retinyl ester counts was similar to that observed in wild-type mice. Plasma triglycerides essentially paralleled the retinol clearance curves over the same time course (data not shown). Further analysis of plasma lipoproteins 2 hrs after gavage showed that the majority of the triglycerides and [^3^H]retinol counts were recovered in the large buoyant lipoproteins of δ<1.006 g/ml (data not shown). These lipoproteins were larger (>100 nm in diameter, Fig. 2B) when compared to samples obtained from wild-type mice, suggesting a defect in the initial lipolysis of triglyceride-rich lipoproteins in the circulation. The rates of triglyceride secretion, measured after injection of Triton WR-1339, were similar in the wild type and mutant (0.032±0.001 mg triglyceride/min in wild type *vs*. 0.032±0.004 mg triglyceride/min in mutant; n = 3, *P* = 1.0) [22], excluding a defect in chylomicron assembly or secretion from the intestine.

To further examine early events in lipoprotein processing, we measured the initial turnover of triglycerides injected intravenously in the form of Intralipid particles containing [^3^H]triolein, which are proposed to resemble chylomicrons [23]. The initial loss of the counts from the plasma was delayed in the mutant (Fig. 2C). Fitting the data to a single exponential decay function yielded fractional clearance rates of 3.2%±0.5/min for the wild type (R^2^ = .96) and 2%±0.5/min (R^2^ = 0.88) for the mutant. In contrast to these findings, hepatic clearance appeared to be normal in *Col18a1* ^−/−^ mice based on clearance of intravenously injected human VLDL (Fig. 2D). Similarly, the rate of clearance of [^3^H]retinol-labeled chylomicrons at later time points was comparable in mutant and wild type (Fig. 2A). Together these data suggest that the hepatic clearance of chylomicrons and VLDL was normal in the mutant. Note that mutants lacking the transmembrane heparan sulfate proteoglycan, syndecan 1, or hepatocyte sulfotransferases involved in heparan sulfate assembly develop hypertriglyceridemia due to defects in hepatic clearance of remnant particles [24], [25], [26]. In contrast, *Col18a1* ^−/−^ mice develop hypertriglyceridemia due to altered extrahepatic clearance triglyceride-rich lipoproteins.

### Lpl expression and activity are normal in *Col18a1* ^−/−^ mice

**Figure 3:**
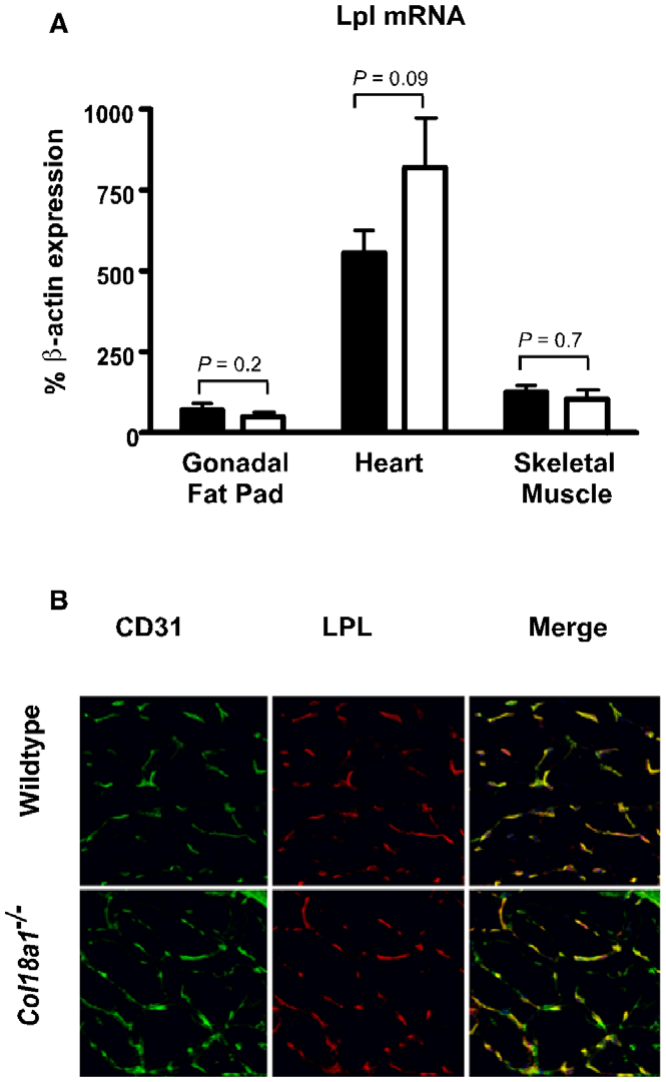
Lpl expression in *Col18a1* ^**−/−**^ mice. (**A**) Lpl mRNA was measured by qPCR in samples prepared from gonadal fat pads, heart and skeletal muscle from mutant and wild-type mice (n = 4 mice per genotype). (**B**) Frozen tissue sections prepared from skeletal muscle from wild-type and *Col18a1* ^−/−^ mice were stained with DAPI and immunohistochemically stained for the endothelial marker CD31 and Lpl. Samples were analyzed by confocal microscopy and compared for differences in the distributions of CD31 (green) and Lpl (red). The distributions of Lpl and CD31 overlap and are not altered in the *Col18a1* ^−/−^ mutant. The images are from one set of samples, but the experiment was repeated on four different mice.

The delayed lipolysis of plasma triglycerides in *Col18a1* ^−/−^ mice could reflect deficiencies in various factors involved in Lpl-mediated turnover, including angiopoietin-like 4 [27], caveolin-1 [28], and apolipoprotein C-II [29]. However, expression of these proteins in skeletal muscle and adipose tissue were unaffected in mutant compared to wild-type mice as measured by qPCR and western blotting (data not shown). Furthermore, Lpl transcripts and proteins in adipose, heart, and muscle tissue were unaffected in both fasted (data not shown) and fed animals (Fig. 3A). Lpl activity in tissue extracts was somewhat higher in mutant vs. wild-type tissues; however, the differences did not reach statistical significance (heart: 1600±300 mU activity/g tissue in wild-type mice vs. 1900±500 in mutants, n = 3, *P* = 0.53; adipose: 48±8 mU activity/g tissue in wild-type mice vs. 65±15 in the mutant, n = 3, *P* = 0.16). Recent studies indicate that Lpl is localized to the vascular endothelium, bound to its receptor, Gpihbp1 [14], suggesting the possibility that Lpl was mislocalized in the mutant. However, immunolocalization studies showed that Lpl distribution was not detectably altered in skeletal muscle from *Col18a1* ^−/−^ mice and colocalized with the blood endothelial cell marker, CD31 (Fig. 3B). The distribution of Lpl was also unaltered in the heart (see below).

**Figure 4:**
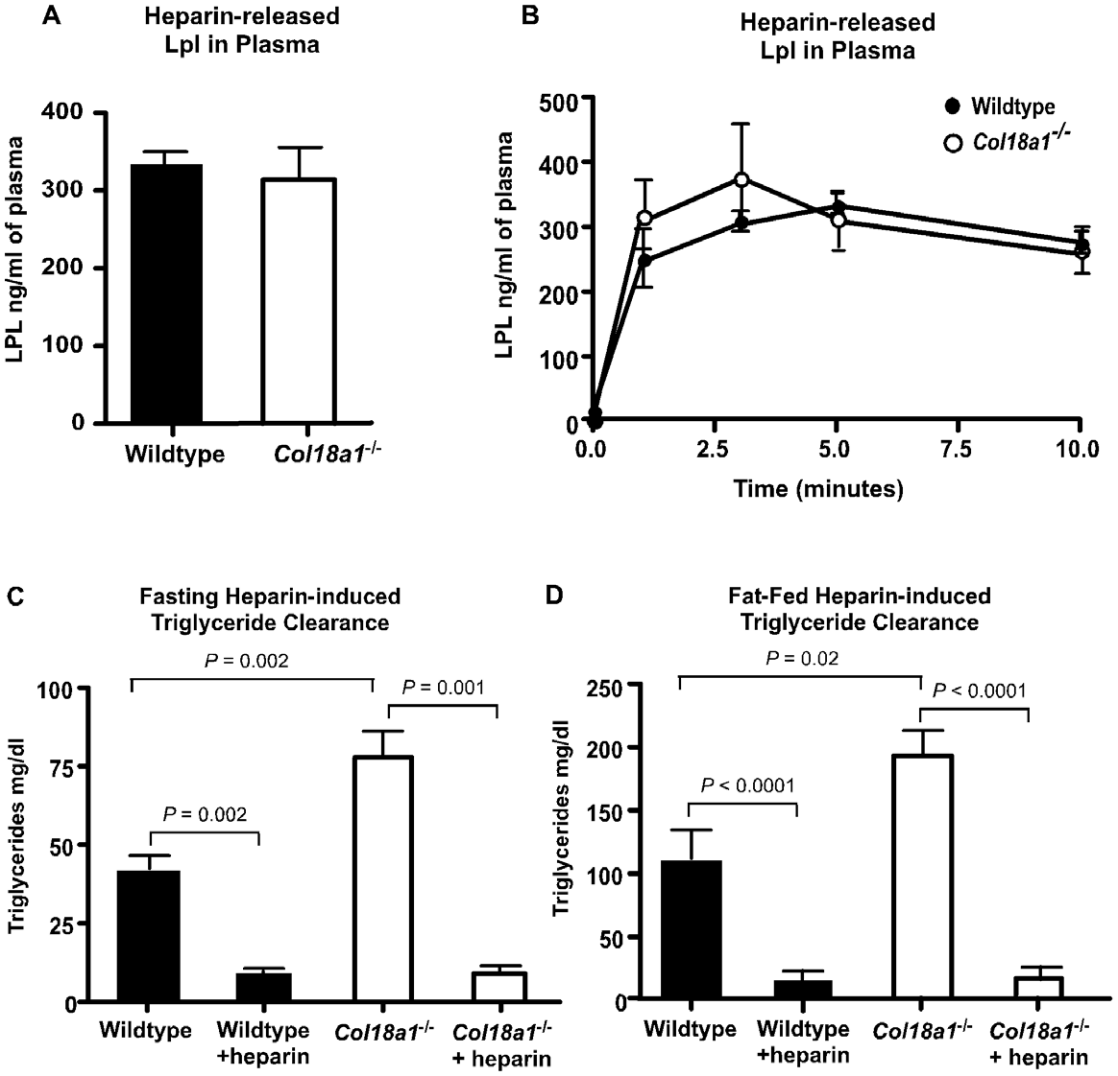
Lpl in *Col18a1^**−/−**^* mice. (**A**) Wild-type and *Col18a1* ^−/−^ mice were injected intravenously with heparin (0.5 U/g body weight) and the levels of Lpl in the plasma were measured 15 min later (n = 7 for each strain, *P* = 0.7). Results are representative of two separate experiments. (**B**) Serial sampling of plasma samples showed similar rates of appearance of Lpl after injection of 0.5 U/g heparin. (**C, D**) Plasma triglycerides were measured pre- and post-heparin injection in wild-type and *Col18a1* ^−/−^ mice after (**C**) a 4-hour fast or (**D**) 2 hours after fat challenge. Heparin was injected intravenously (0.5 U/g body weight) and blood was sampled from the retro-orbital sinus 10 minutes after injection. Results were verified in two independent assays.

To test if Lpl could be mobilized in the mutant, animals were intravenously injected with 0.5 U of heparin. Twenty minutes after injection, plasma Lpl levels were comparable in mutant and wild-type mice (Fig. 4A; *P* = 0.7). The time course of release of Lpl was also comparable (Fig. 4B). Furthermore, heparin administration resulted in the rapid disappearance of plasma triglycerides in *Col18a1* ^−/−^ and wild-type mice under fasting conditions (Fig. 4C) as well as in mice challenged orally with corn oil (Fig. 4D). Thus, the mobilization of Lpl was normal in the mutant and the triglyceride-rich lipoproteins that accumulated under fasting and post-prandial conditions were fully capable of undergoing lipolysis when exposed to Lpl.

### Lumenal Lpl is reduced in *Col18a1* ^−/−^ mice

**Figure 5:**
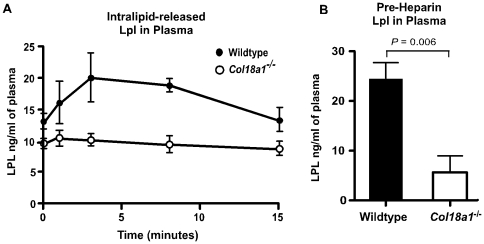
Pre-heparin plasma Lpl was reduced in *Col18a1* ^**−/−**^ mice. (**A**) Mice were injected intravenously with a bolus of Intralipid to mimic hyperchylomicronemia and plasma Lpl was measured at the times indicated (*n* = 4 for each strain; *P*<0.004). Results are representative of two separate experiments. The areas under the curves were 75 ng/ml in wild type vs. 8.2 ng/ml in *Col18a1* ^−/−^ mice. (**B**) Plasma Lpl was measured by sandwich ELISA in wild-type (black bars) and *Col18a1^−/−^* mice (white bars) (n = 7 for each strain; *P* = 0.006).

Reduced presentation of Lpl at the lumenal side of capillaries could explain the phenotype of *Col18a1* ^−/−^ mice [30]. To explore this possibility, we injected mice with Intralipid, which unlike heparin displaces only Lpl bound to the luminal surface of the endothelium [15], [31]. In wild-type mice, Intralipid induced the release of Lpl into the plasma (Fig. 5A), albeit at much lower levels than heparin (Fig. 4A). In contrast, mutant mice did not exhibit any significant increase in plasma Lpl after injection. After correction for the normal plasma pool of Lpl, the areas under the curves showed a dramatic difference in displaceable enzyme in the mutant (75 ng/ml in wild type vs. 8.2 ng/ml in mutant, *n* = 8 in two separate experiments). Since Lpl is rapidly cleared in the liver [32], [33], this technique may underestimate the amount of Lpl bound to the endothelium; nevertheless, the levels are significantly lower in *Col18a1^−/−^* mice compared to wild-type mice.

Inspection of the curves in Fig. 5A showed that the baseline level of plasma Lpl (t = 0) was lower in the mutant. To avoid any effect of preparing the animals for injection, we analyzed Lpl levels in plasma of naive animals. As shown in Fig. 5B, the steady-state level of plasma Lpl was much lower in the mutant (5±4 ng of Lpl/mL in the mutant vs. 24±4 ng Lpl/mL in the wild type, *P* = 0.006). Attempts to measure enzyme activity in the plasma were not successful because the signal-to-noise ratio for the assay was not sufficient, even after enrichment of plasma samples by heparin-Sepharose chromatography.

**Figure 6:**
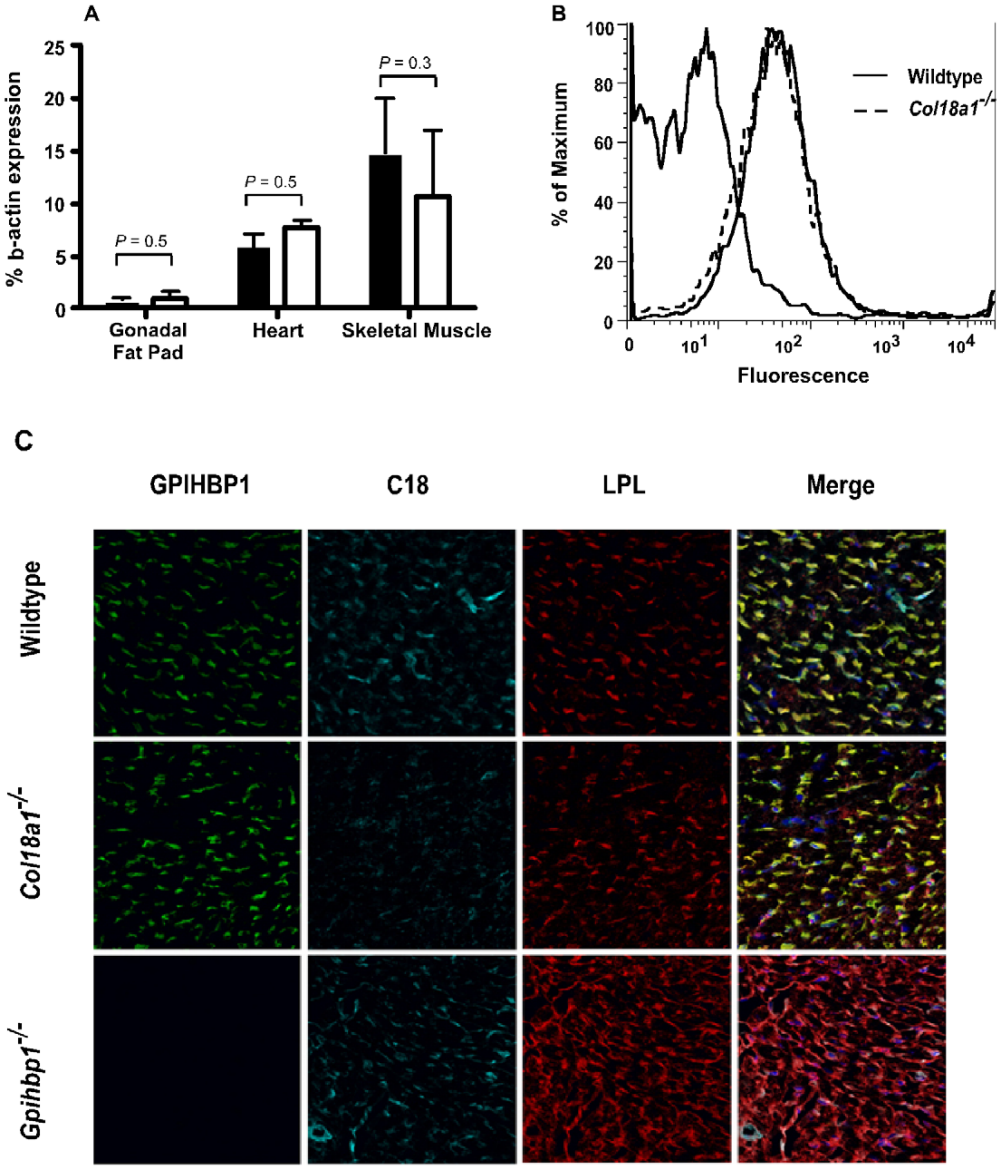
Normal vascular distribution of Gpihbp1 in *Col18a1* ^**−/−**^ mice. (**A**) mRNA expression was determined in RNA samples isolated from gonadal fat pad, heart and soleus muscle from wild-type and *Col18a1* ^−/−^ mice (n = 4, for each strain). Results were verified in two independent assays. (**B**) Primary cardiac endothelial cells were isolated from control (solid line) and *Col18a1* ^−/−^ mice (dashed line) by collagenase digestion and magnetic beads separations using anti-CD31 antibodies. Gpihbp1 was detected with a specific polyclonal antibody and Alexa-488 labeled anti-rabbit secondary antibodies followed by FACS analysis. The curve to the left represents cells incubated with a control IgG and secondary antibodies. Data are representative of 3 separate experiments. (**C**) Cardiac tissue sections from wild-type, *Col18a1* ^−/−^ and *Gpihbp1* ^−/−^ mice were analyzed by confocal microscopy and compared for differences in the distributions of Lpl (red), Col18 (blue) and the Gpihbp1 (green). The distributions of Lpl and Gpihbp1 overlap with Col18 staining in wild-type heart tissues and are not altered in the *Col18a1* ^−/−^ mutant. The patterns of Lpl and Gpihbp1 staining were unaffected in skeletal muscle and fat (data not shown).

Mice lacking Gpihbp1 also have markedly reduced amounts of lumenally-exposed Lpl [14], suggesting the possibility that Gpihbp1 expression might be reduced in *Col18a1* ^−/−^ mice. However, transcript levels of *Gpihbp1* were comparable in mutant and wild-type mice in adipose, heart, and muscle (Fig. 6A). Flow cytometric analysis of freshly isolated cardiac endothelial cells using an anti-mouse Gpihbp1 antibody showed no difference in expression (Fig. 6B). Furthermore, confocal microscopy demonstrated that Gpihbp1, Col18 and Lpl have a similar vascular distribution pattern in cardiac tissue sections of *Col18a1* ^−/−^ and wild-type mice (Fig. 6C). However, the distribution of Lpl was mislocalized in *Gpihbp1* ^−/−^ mice as recently reported [14].

### Hypertriglyceridemia occurs in Knobloch patients lacking the vascular form of Col18

The hypertriglyceridemia and reduction in plasma and endothelium-bound Lpl in *Col18a1^−/−^* mice prompted further evaluation in humans with Col18-deficiency. In humans, loss of function mutations in *COL18A1* cause Knobloch syndrome (OMIM 267750). This extremely rare disorder is characterized primarily by ocular defects, including myopia, vitreoretinal and macular degeneration, retinal detachments, and occipital encephalocele defects thought to arise from altered basement membrane structures [34], [35], [36], [37], [38], [39], [40]. Several of the ocular defects are similar to defects observed in *Col18a1* ^−/−^ mice [8].

**Figure 7:**
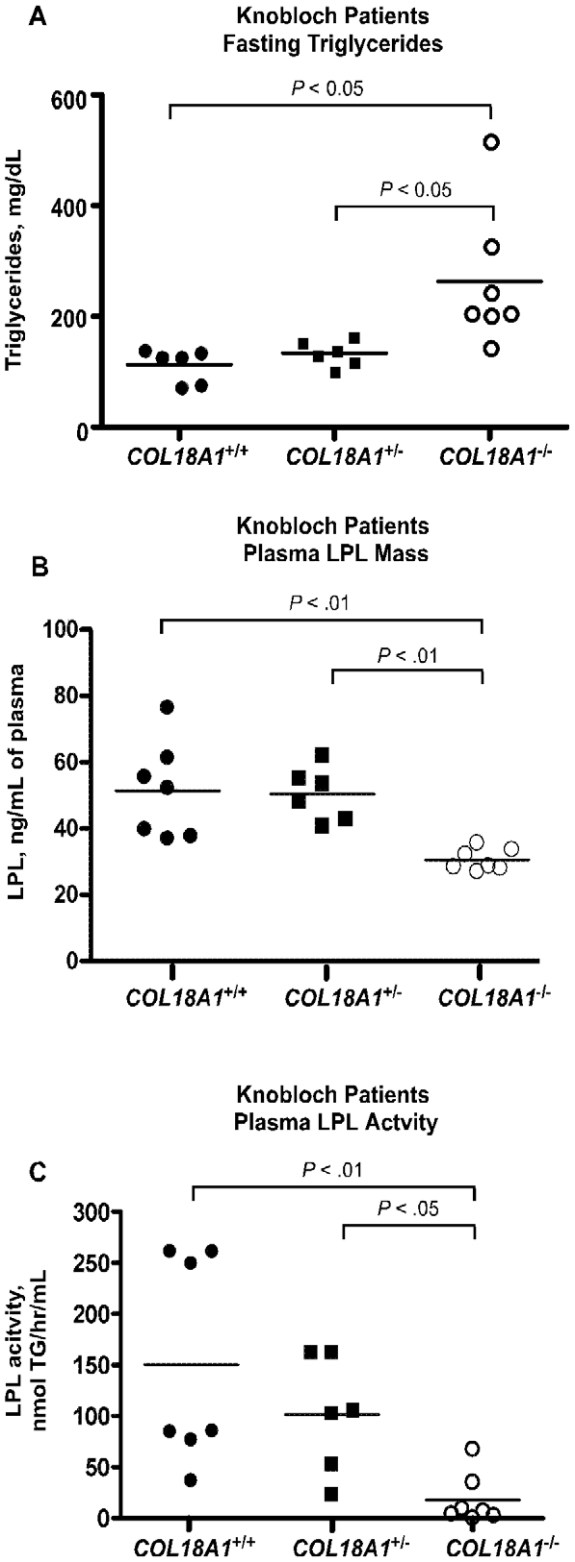
Hypertriglyceridemia in Knobloch patients. (**A**) Plasma triglycerides were measured in blood samples collected from patients with Knobloch syndrome (*COL18A1*−/−) (open circles, n = 7), heterozygous carriers (*COL18A1^+/−^*, closed squares, n = 6) and normal control subjects (*COL18A1^+/+^*, closed circles, n = 6). Plasma triglycerides differed by group (*P* = 0.011) and the difference between Knobloch and normals was significant (P<0.05) (**B**) Plasma Lpl levels were measured by sandwich ELISA in previously frozen heparinized plasma collected from fasting subjects with Knobloch syndrome (open circles), heterozygous carriers (closed squares) and normal control family members (closed circles). Plasma Lpl levels differed by group (*P* = 0.001) and Knobloch levels were reduced compared to heterozygotes (*P*<0.01) and unaffected family members (*P*<0.01) (**C**) Activities levels of Lpl were measured according to the assay described in Methods and converted to units of (nanomoles) triglyceride hydrolyzed per hour per ml plasma. Knobloch plasma contained reduced Lpl activity compared to heterozygous carriers (*P*<0.05) and normal family members (*P*<0.01).

A large consanguineous family of Knobloch patients was identified in a remote farming community in Bahia State, Brazil [39]. Linkage analysis studies showed that this family carries a null mutation predicted to disrupt specifically the NC11-303 isoform (short) of *COL18A1* common to vascular basement membranes, which is distinct from a long form splice variant expressed abundantly in the liver and heart and regulated by a different promoter [41]. Although small in number, the members of this rare family constituted an ideal study group to test if a more specific deficiency in vascular Col18 might give rise to elevated plasma triglycerides as well. Normal, heterozygous, and homozygous individuals had similar diets, shared living quarters, and were selected with similar ages. Plasma cholesterol, glucose, and triglyceride levels in plasma samples taken from seven Knobloch patients were compared to six heterozygous carriers of the mutation and seven normal controls (see Methods for study inclusion criteria Supplemental Table S1). Fasted plasma triglycerides were elevated to >200 mg/dl in 6 of 7 affected Knobloch patients compared to <150 mg/dl in unaffected family members (*P*<0.05), but no significant difference was noted in heterozygotes compared to normal or affected subjects (Fig. 7A). Ultracentrifuge separation and analysis showed that the triglycerides accumulated in lipoproteins of δ<1.006 g/ml (data not shown). Plasma cholesterol was elevated slightly, but the difference did not achieve statistical significance with the limited number of individuals. One of the patients (F1020-36, Supplemental Table S1) was borderline diabetic (blood glucose = 130 mg/dl) in a field measurement, but was euglycemic when tested again at a later time by colorimetric assay. Thus, the hypertriglyceridemia seen was not due to diabetes or other diseases that were diagnosed by their attending physician. A single plasma sample from another Knobloch patient from the same family, who was not included in Table S1 but lived in Sao Paulo, Brazil, was compared to four unaffected household members with similar results (plasma triglycerides 529 mg/dl in the affected patient *vs*. 232±48 mg/dl in controls, n = 4).

Finally, fasting plasma samples from Knobloch, heterozygous and normal control related family members were compared for differences in Lpl mass and activity levels. Results demonstrated that the plasma levels of LpL enzyme in Knobloch subjects were reduced compared to related heterozygous and normal control family members (Fig. 7B, 31±3 ng/ml for Knobloch patients, 50±8 for heterozygotes, *P*<0.01; and 51.4±15 for normal family members, *P*<0.01). Unlike mouse plasma, human plasma had sufficient Lpl activity to measure reliably. Knobloch patients also exhibited reduced plasma Lpl activity (Fig. 7C, 18±9 nmol triglycerides hydrolyzed/hr/ml; 101±56 for heterozygotes, *P*<0.05; and 151±101 for the wild type, *P*<0.01). The extreme remote location of most family members and local dietary restraints precluded other postprandial studies and interventions.

## Discussion

This report demonstrates that Col18-deficiency results in elevated plasma triglycerides and low plasma Lpl abundance and activity. To our knowledge, this is the first demonstration of a requirement for a specific extracellular matrix heparan sulfate proteoglycan in triglyceride metabolism *in vivo*. This function was unique to Col18 and not associated with perlecan, another basement membrane heparan sulfate proteoglycan. Previous studies showed that altering the expression of a specific sulfotransferase involved in the formation of heparan sulfate chains in hepatocytes and syndecan-1, a transmembrane heparan sulfate proteoglycan, affected hepatic clearance of triglyceride-rich lipoproteins [24], [25], [26]. In contrast, the defect in *Col18a1^−/−^* mice appears to be due to reduced lipolysis of triglyceride-rich lipoproteins in the peripheral circulation due to decreased presentation of Lpl in the plasma compartment. Data supporting this conclusion derive from the large size of triglyceride-rich lipoproteins that accumulate in the mutant under fasting and postprandial conditions, differences in the initial turnover of [^3^H]retinol-labeled lipoproteins, and the lack of any effect on hepatic turnover of remnant lipoproteins derived from dietary triglyceride and injected human VLDL. Confirmatory evidence was provided by studies of human Knobloch patients bearing a mutation that affects the vascular short isoform of Col18.

How does a proteoglycan located in basement membranes affect presentation of Lpl in the plasma and at the endothelial surface? Lpl is secreted by the parenchymal cells of adipose, cardiac and skeletal muscle and migrates from its site of secretion through the interstitial space, across basement membranes and eventually the endothelium [12]. Because Lpl contains one or more heparin binding sites [17], [42], [43], [44], it presumably can interact with the heparan sulfate chains on Col18 or other matrix proteoglycans [10]. Binding to Col18 could facilitate diffusion or protect Lpl during its transport to the endothelial surface, perhaps in the same way that heparan sulfate proteoglycans have been suggested to affect the rate of diffusion or turnover of heparan sulfate-binding morphogens during early development (*e.g.*, Wnts, Hedgehog, FGFs, BMPs) [45], [46], [47], [48]. The inefficient presentation of Lpl could also reflect the expansion and thickening of basement membranes that occurs in various tissues (1.4 to 2-fold) due to Col18 deficiency [11]. A quadratic relationship exists between time and distance for freely diffusing molecules [49], therefore, small changes in distance could greatly slow the rate of diffusion of Lpl. In either model, the delay in transport could decrease the rate at which the active enzyme appears in the blood compartment without significantly affecting total tissue Lpl as measured by mobilization by heparin. Since Lpl is displaced from Gpihbp1 by circulating triglyceride-rich lipoproteins and rapidly cleared in the liver [15], a decrease in its rate of diffusion through tissues could diminish the steady-state amount of active enzyme bound to Gpihbp1 on apical endothelium and in the plasma. Although simple in concept, proving this model has proven to be difficult due to the absence of suitable in vitro models for measuring transport through tissues and across vascular basement membranes.

Interestingly, the phenotype of *Col18a1^−/−^* mice resembles that seen in mutants that affect Lpl expression or presentation. For example, Lpl heterozygotes have reduced plasma levels of Lpl, mild hypertriglyceridemia in both the fasted and post-prandial states, and acute accumulation of plasma lipids after oral gavage [50]. Gpihbp1-deficient animals also display decreased plasma Lpl and Lpl associated with the endothelium (i.e. Lpl displaceable by Intralipid) and injection of heparin raises plasma Lpl to the same levels as the wild type [15]. However, *Gpihbp1* ^−/−^ mice differ significantly from *Col18a1* ^−/−^ mice in the extent of hypertriglyceridemia and localization of Lpl in tissues [14], [15]. In *Col18a1* ^−/−^ mice, Lpl colocalized with CD31^+^ endothelium (Fig. 3), but it was absent on the apical endothelium of brown fat capillaries of *Gpihbp1* ^−/−^ mice, when the capillaries were viewed in cross-section. The abundance of Lpl was not altered in *Col18a1* mutants, but it was increased in extracellular matrix that extended beyond the distribution of endothelial and vascular basement membrane markers in capillaries of *Gpihbp1* mutants. Presumably these differences reflect not only the amount of Lpl bound to the endothelium, but also the rate at which it transfers from the subendothelium to the lumenal side of the vasculature.

Recent large cohort clinical trials have shown that mildly elevated triglycerides are a major risk factor for coronary heart disease [51], [52]. The work presented here shows that disruption of Col18 in humans is associated with an increase in plasma triglycerides, suggesting that decreased Col18 expression or mutations affecting its heparan sulfate chains might account for some cases of hypertriglyceridemia. For example, patients with diabetes mellitus exhibit delayed metabolism of dietary triglycerides in post-prandial studies [53], [54]. The direct cause of postprandial hyperlipidemia in these patients remains unknown, but it is interesting to note that thickening of basement membranes has been documented in the intima of the aorta as well as muscle, glomerular and retinal capillaries in diabetes [55], [56].

## Methods

### Mice and animal husbandry

Mice deficient in Col18 (*Col18a1* ^−/−^), and perlecan (*Hspg2 ^Δ3/Δ3^*) were described previously [8], [9], [10], [57], [58], [59]. All mice were backcrossed >10 generations on a C57Bl/6 background and housed in vivaria approved by the Association for Assessment and Accreditation of Laboratory Animal Care located in the School of Medicine, University of California, San Diego, following standards and procedures approved by the local Institutional Animal Care and Use Committee (protocol S99127). Mice were weaned at 3 weeks, maintained on a 12 hr light-dark cycle, and fed *ad libitum* with water and standard rodent chow (Harlan-TekLad) unless indicated otherwise. Genotyping of *Col18A1* ^−/−^ was performed as described [8].

### Lipoprotein analysis

Plasma was prepared from retroorbital bleeds after fasting the animals for 4 hr in the morning and plasma triglyceride and cholesterol levels were determined enzymatically (Wako). Pooled mouse plasma lipoproteins were also separated by gel filtration FPLC (LipoSEARCH, Skylight Biotech Inc.). Plasma samples were centrifuged for 20 hr at 45,000× rpm in a Beckman 50.3Ti rotor at δ = 1.006 g/ml to collect triglyceride-rich lipoproteins. Isolated lipoproteins were dialyzed against PBS and analyzed for lipid content.

### Electron microscopy

Lipoproteins of δ<1.006 g/ml were purified from pooled plasma obtained from fasted animals or after oral gavage of 0.2 ml corn oil (n = 3 or each genotype). To determine particle size, samples were coded, negatively stained with 2% potassium phosphotungstate (pH 7.6), and imaged by transmission electron microscopy (Jenny Wong, Gladstone Institute). The diameters of particles in 8 fields were measured, and the data from two separate experiments were pooled.

### Triglyceride secretion rates in mice

Triglyceride secretion rates were determined by the method of Hirano et al. [22]. Briefly Triton WR-1339 (500 mg/kg body weight, Sigma) was injected *via* the tail vein into mice fasted for 4 hr, and triglycerides were measured in plasma samples taken 30, 60, and 90 min post-injection. The secretion rate (mg/min) was calculated from the increment in triglyceride concentration per minute multiplied by the plasma volume of the mouse (estimated as 0.035% of body weight in grams). To determine the intestinal triglyceride secretion rate, Triton WR-1339 was injected into mice that had been given a bolus of corn oil by gavage (0.2 ml/mouse).

### VLDL, Intralipid, and retinol clearance

Clearance of human VLDL was performed by measuring the level of human apoB-100 present in plasma at timed intervals following intravenous injection of VLDL purified from fasted, healthy human donors. Human apoB-100 was measured by ELISA using mAb MB47, which binds human but not murine apoB-100, exactly as described in [24]. [^3^H]triolein-labeled Intralipid particles were made as described previously [23]. Samples (5×10^5^ cpm) were injected into the tail vein, blood samples were taken at the indicated times, and radioactivity was measured (10 µl of serum) by scintillation counting. Retinol excursion studies were done essentially as described [60]. Briefly, 27 µCi of [11,12-^3^H]retinol (Perkin-Elmer, 44.4 Ci/mmol) in ethanol was mixed with 1 ml of corn oil (Sigma) and administered by oral gavage (200 µl/mouse). Blood was sampled at the times indicated by retro-orbital sinus bleed and radioactivity was measured in triplicate (10 µl of serum) by scintillation counting.

### mRNA expression

Lpl and Gpihbp1 mRNA expression were determined in RNA samples isolated from gonadal fat pad, heart and soleus muscle from wild-type and *Col18a1* ^−/−^ mice (n = 4, for each strain). RNA was isolated (10 mg/tissue, Trizol reagent), reverse transcribed (Superscript III, Invitrogen) and amplified using gene specific intron spanning primers to Lpl or Gpihbp1. Primer sequences, Lpl, 5′-AGGTGGACATCGGAGAACTG and 3′- TTTGTCCAGTGTCAGCCAGA and, Gpihbp1, 5′- AACATGATCCCTGGAAGCAG and 3′- ACAGTGTGGACTGGCAACAG. Quantitation was done by the 2^−ΔΔCt^ method using β-actin as a control RNA in a Stratagene MxP3500. Final numbers represent fold-expression compared to β-actin (100%). Ct values from triplicate assays were used to calculate fold-expression according to Stratagene manual. Results were verified in two independent assays.

### Lipoprotein lipase activity and protein levels

Intravenous injection of heparin (0.5 U/g body weight) or 20% Intralipid (7.5 ml/kg body weight) was used to release Lpl into the plasma. Blood samples were taken by retro-orbital bleeding prior to and at various times after injection. Lpl mass in plasma samples was measured by ELISA with a specific antibody as described previously [61].

Lpl activity in plasma or tissues was determined using sonicated radiolabeled triolein substrate (tri[9,10(n)-^3^H]olein, ∼0.5 mCi/ml; Perkin Elmer) and calculated as nmol triolein hydrolyzed per min (*i.e.* mU activity) for each sample [62]. Levels of human Lpl were determined by sandwich enzyme-linked immunosorbent assay with a monoclonal anti-human Lpl antibody and polyclonal anti-Lpl antibody (ALPCO Diagnostics, Salem, NH) according to standard methods [63]. Levels of Lpl mass in plasma of mice was determined by ELISA with immunopurified goat antibodies against mouse Lpl [61].

### Immunohistochemical localization of Lpl

Mice were perfused through the left ventricle with ∼10 ml phosphate-buffered saline at a rate of 2–3 ml/min after cutting the inferior vena cava, then perfused with 10 ml 3% paraformaldehyde in PBS. Excised tissues were post-fixed in 3% paraformaldehyde for 1 hr at room temperature, placed in 30% sucrose dissolved in PBS overnight at 4°C and embedded the next day in OCT.

To detect LPL in mouse tissues, 10 µm-thick frozen sections were prepared and incubated overnight with primary antibodies [1∶400 for the hamster anti-CD31 antibody (Millipore, Billerica, Mass), 1∶800 for the rat anti-GPIHBP1 antibody [64], 1∶500 for the rabbit anti-collagen type XVIII antibody (Cosmo Bio USA, Carlsbad, CA), and 10 µg/ml for the goat anti–mouse LPL antibody]. Secondary antibodies (Alexa488- or Alexa549-labeled anti-hamster IgG, Alexa649-labeled anti-rat IgG, Alexa488- or Alexa649-labeled anti-rabbit IgG, and Alexa555-labeled anti-goat IgG) were used at a dilution of 1∶200 and were incubated with slides at room temperature for 1–2 h. Images were obtained by confocal fluorescence microscopy with a Leica SP2 1P-FCS microscope (Heidelberg, Germany). Images along the z-axis were captured sequentially with a 63× objective and merged images were generated with the Volocity 3D rendering software (version 4.4; PerkinElmer Improvision).

### Human Subjects

Knobloch patients were recruited from a large family diagnosed with a loss of function mutation affecting the expression of the vascular isoform of *COLA18* and characterized previously at the Human Genome Center in São Paulo, SP, Brazil [39]. Most members of this large family live in a remote farming community in Bahia State, Brazil and family members typically had similar diets and shared living quarters. Approval for human studies and informed consent of subjects were obtained from the Ethical Committee of the Institute of Biosciences, University of São Paulo (São Paulo, Brazil) and human studies were approved by the University of California, San Diego, Human Subjects Protection Program, #091434X. After obtaining signed informed consent, a study investigator obtained a medical history and patients were fasted for 4 hr after which a plasma sample was taken. Glucose measurements were taken immediately with a glucometer. Study participants with >200 mg/dl blood glucose, previously diagnosed diabetes, heavy alcohol consumption (5 alcoholic drinks per day), and tobacco use were excluded from the study (4 total). Plasma triglyceride, cholesterol, and glucose levels from 7 homozygous patients, 6 heterozygotes, and 6 age-matched normal subjects from the family were measured using kits (Wako). A second affected family member living in São Paulo, Brazil along with 4 wild-type controls living in the same household was tested.

### Statistics

Statistical analyses were performed using PRISM (GraphPad Software). All data are expressed as mean values ± SD unless otherwise indicated. Comparisons between wild-type and *Col18a1^−/−^* mice were determined using an unpaired Student's (two-tailed) t test. Knobloch, heterozygote carriers and unaffected family members were compared by the non-parametric Kruskal-Wallis test with Dunns post-test comparisons between all pairs of genotypes. Significance was taken as *P*<0.05.

## Supporting Information

Table S1 Knobloch patient data.
(0.05 MB DOC)
