# Supplementary material for: Deletion of the Basement Membrane Heparan Sulfate Proteoglycan Type XVIII Collagen Causes Hypertriglyceridemia in Mice and Humans
Source: PLoS One. 2010 Nov 10;5(11):e13919. doi: 10.1371/journal.pone.0013919 (PMC2978080; doi:10.1371/journal.pone.0013919)
Supplement: Table S1 — Knobloch patient data. (0.05 MB DOC) [file pone.0013919.s001.doc]

# Supplemental Table S1: Knobloch patient data.

| Patient I.D | Col18 genotype | fasting glucose mg/dl | fasting triglycerides  mg/dl | fasting cholesterol mg/dl |
| --- | --- | --- | --- | --- |
|  |  |  |  |  |
| F1020-03 | +/+ | 91 | 144.1 | 107.5 |
| F1020-17 | +/+ | 83 | 139.3 | 81.4 |
| F1020-39 | +/+ | 91 | 118.9 | 86.6 |
| F1020-45 | +/+ | 89 | 130.6 | 79.9 |
| F1020-46 | +/+ | 72 | 104.8 | 85.8 |
| F1020-50 | +/+ | 86 | 133.8 | 84.0 |
| F1251-04 | +/+ | 85 | 118.8 | 118. |
| mean |  | 85 ± 6 | 117 ± 12 | 92 ± 15 |
|  |  |  |  |  |
| F1020-05 | +/- | 82 | 169.6 | 74.6 |
| F1020-40 | +/- | 89 | 144.4 | 90.7 |
| F1020-47 | +/- | 75 | 143.8 | 92.9 |
| F1020-46 | +/- | 72 | 104.8 | 85.8 |
| F1020-48 | +/- | 85 | 133.5 | 86.9 |
| F1020-54 | +/- | 103 | 81.9 | 71.6 |
| mean |  | 84 ± 11, *P* = 0.85 | 135 ± 9, *P* = 0.27 | 84 ± 9, *P* = 0.43 |
|  |  |  |  |  |
| F1020-06 | -/- | 92 | 540.1 | 107.9 |
| F1020-09 | -/- | 87 | 205.4 | 107.5 |
| F1020-35 | -/- | 95 | 248.4 | 93.3 |
| F1020-34 | -/- | 89 | 122.9 | 122.4 |
| F1020-36 | -/- | 130* | 330.6 | 101.1 |
| F1020-37 | -/- | 82 | 210.9 | 75.0 |
| F1020-38 | -/- | 93 | 209.4 | 82.5 |
| mean |  | 95 ± 15, *P* = 0.14 | 267 ± 51, *P* = 0.02 | 99 ± 16, *P* = 0.26 |
|  |  |  |  |  |

# Fasted blood samples were taken from Knobloch patients.

# Wt – wildtype, +/-, heterozygous carriers of Col18 mutation, -/-, Knobloch Syndrome, homozygous Col18 mutation. * borderline diabetic. All P values compared to wildtype.
